# Molecular dynamics of bicyclo[2.2.0]hex-2-ene ring opening and its polar derivative: allowed *vs.* forbidden pathways

**DOI:** 10.1039/d5sc07711g

**Published:** 2026-02-10

**Authors:** Zhixin Qin, Qingyang Zhou, Rong-Kai Wu, K. N. Houk

**Affiliations:** a SINOPEC (Beijing) Research Institute of Chemical Industry Co. Ltd Beijing 100013 China; b Department of Chemistry and Biochemistry, University of California Los Angeles California 90095-1569 USA houk@g.ucla.edu; c Center of Chemistry for Frontier Technologies, Department of Chemistry, Zhejiang University Hangzhou 310027 China

## Abstract

We employed density functional theory, CCSD(T) and CASSCF computations, along with quasi-classical molecular dynamics simulations, to explore the ring opening of bicyclo[2.2.0]hex-2-ene and its 1-amino-4-cyano derivative. While the overall reaction is a formally forbidden 4-electron disrotatory electrocyclization, both conrotatory and disrotatory pathways operate for the hydrocarbon, the latter involving a HOMO–LUMO crossing and diradical transition state. Quasi-classical simulations reveal the presence of non-statistical dynamic behavior involving a short-lived intermediate in the formally forbidden process. For the donor/acceptor-substituted derivative, the charge separation induced by substitution eliminates orbital symmetry restrictions, enabling a sterically favored disrotatory pathway.

## Abstract 2

We employed density functional theory, CCSD(T) and CASSCF computations, along with quasi-classical molecular dynamics simulations, to explore the ring opening of bicyclo[2.2.0]hex-2-ene and its 1-amino-4-cyano derivative.

## Graphical abstract

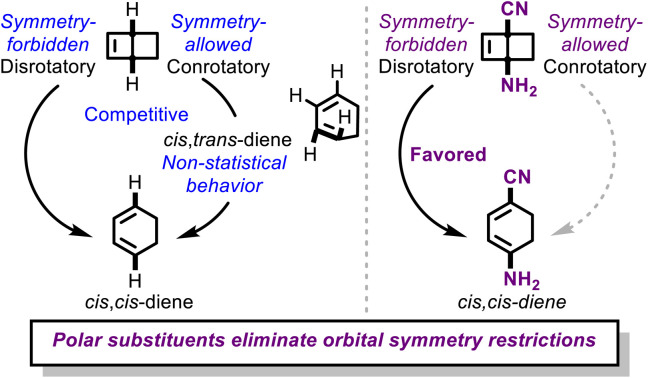

## Introduction

**Scheme 1:**
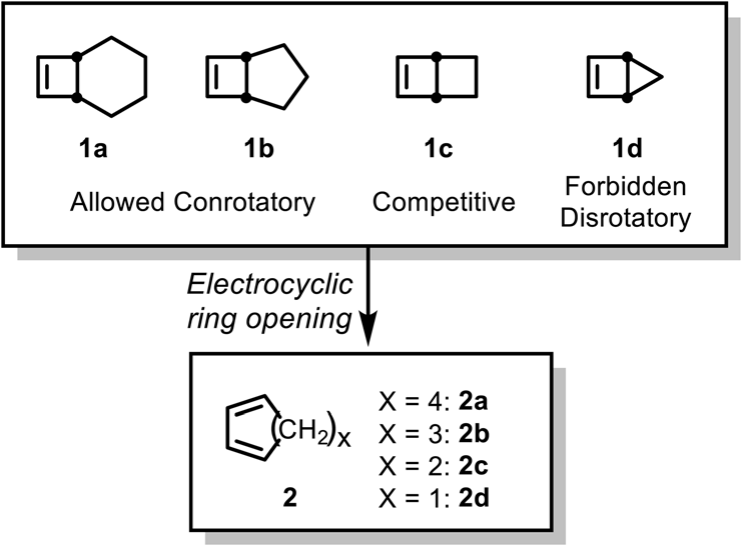
Electrocyclic ring opening of bicyclo[*X*.2.0]alkenes based on the computational results of de Lera *et al.*^5,6^

The electrocyclic ring opening of bicyclo[2.2.0]hex-2-ene and its analogs has been studied extensively, both experimentally and computationally.^1–4^ The significant ring strain in these systems enhances the formally forbidden disrotatory 4-electron electrocyclization. In 1976, Goldstein *et al.* measured the activation parameters for the thermal electrocyclic ring opening of bicyclo[2.2.0]hex-2-ene (Δ*H*^‡^ = 32.2 ± 0.09 kcal mol^−1^) and confirmed the simple electrocyclization mechanism using isotope labeling.^1^ De Lera *et al.* further explored a series of bicyclo[*X*.2.0]alkenes using various DFT and wavefunction methods.^5,6^ Bicyclo[4.2.0]oct-7-ene (1a) first undergoes an allowed conrotatory electrocyclization to give the strained *cis*,*trans-*diene, which then undergoes an *E*–*Z* isomerization to form the final product (Scheme 1). Computed kinetic isotope effect (KIE) values for conrotatory TSs were reported to be in good agreement with the experimental values.^7,8^ Similarly, bicyclo[3.2.0]hept-6-ene (1b) was found computationally to follow the same mechanism, while the smallest analog, bicyclo[2.1.0]pent-2-ene (1d) undergoes only the forbidden disrotatory process. The barriers for the allowed and forbidden pathways for bicyclo[2.2.0]hex-2-ene (1c) were predicted to be competitive.

Woodward–Hoffmann rules predict that 4-electron electrocyclic reactions should proceed exclusively *via* conrotatory pathways.^9,10^ Indeed, for larger rings like bicyclo[3.2.0]hept-6-ene (1b), UV photoelectron spectroscopy confirms adherence to this rule.^11^ However, for smaller analogs such as bicyclo[2.1.0]pent-2-ene (1d) and bicyclo[2.2.0]hex-2-ene (1c), geometric constraints override these orbital symmetry rules. The “allowed” conrotatory pathway would yield highly strained *trans*-cyclic products. Consequently, the reaction is forced to follow the formally forbidden disrotatory pathway—a scenario explicitly anticipated by Woodward and Hoffmann.^9,10^ Beyond these geometric constraints, our group previously demonstrated that electronic effects—specifically, polar substitutions—can also eliminate orbital symmetry restrictions by stabilizing zwitterionic transition states.^12,13^

In this work, we employed ωB97X-D functional and CCSD(T) calculations to explore the electronic mechanisms and dynamics of the disrotatory and conrotatory pathways. Our results reveal that the symmetry-allowed, yet sterically constrained, conrotatory pathway remains closed-shell throughout, whereas the forbidden disrotatory pathway proceeds *via* a diradical transition state.^14^ The calculated free energy barriers indicate that the allowed conrotatory ring opening occurs at approximately the same rate as the forbidden process, as de Lera^5^ and Carpenter^15^ noted in previous studies.

We further investigated these reactions using molecular dynamics simulations to probe potential non-statistical behavior. Previous studies by Carpenter,^16–23^ Singleton,^24–33^ Houk,^34–38^ and others^39–46^ have extensively characterized non-statistical dynamics, often involving diradicals with low barriers for subsequent reactions. Carpenter pioneered in the discussion of such potential energy surfaces in 1990, noting at the time that full-scale trajectory calculations were impractical with modern supercomputers for molecules of interest to organic chemists.^23^ With the advent of high-performance computing, he later realized these simulations, exploring reactions where multiple products arise from diradical intermediates and identifying inertial effects as the source of non-statistical behavior.^16–23,47^ Singleton has similarly characterized dynamic control in bifurcating cycloaddition surfaces,^31^ and Houk's group has reported related effects for thermal and photochemical reactions.^34–38^

Using quasiclassical molecular dynamics (MD) simulations, we reveal that the conrotatory ring opening of bicyclo[2.2.0]hex-2-ene exhibits non-statistical dynamic behavior. Specifically, the *cis*,*trans*-cyclohexadiene possesses a lifetime significantly shorter than predicted by transition state theory (TST).

We also investigated the highly polar 1-amino-4-cyano derivative. In previous work, we discovered that polarization of the hydrocarbon, vinylidenesesquifulvalene studied experimentally by Prinzbach follows the orbital-symmetry-forbidden conrotatory electrocyclic pathway for the 14-electron system.^12^ We showed that system, as well as some hypothetical donor–acceptor 6-electron systems, are no longer governed by the Woodward–Hoffmann rules due to the zwitterionic character of the substrates and transition states.^12^ We now report the effect of donor–acceptor substitution on bicyclo[2.2.0]hex-2-ene. We report for the first time that the donor–acceptor substitution on a 4-electron system leads to a facile concerted, but W–H forbidden, disrotatory pathway due to a zwitterionic transition state that eliminates orbital symmetry restrictions.^12^

## Results and discussion

**Fig. 1:**
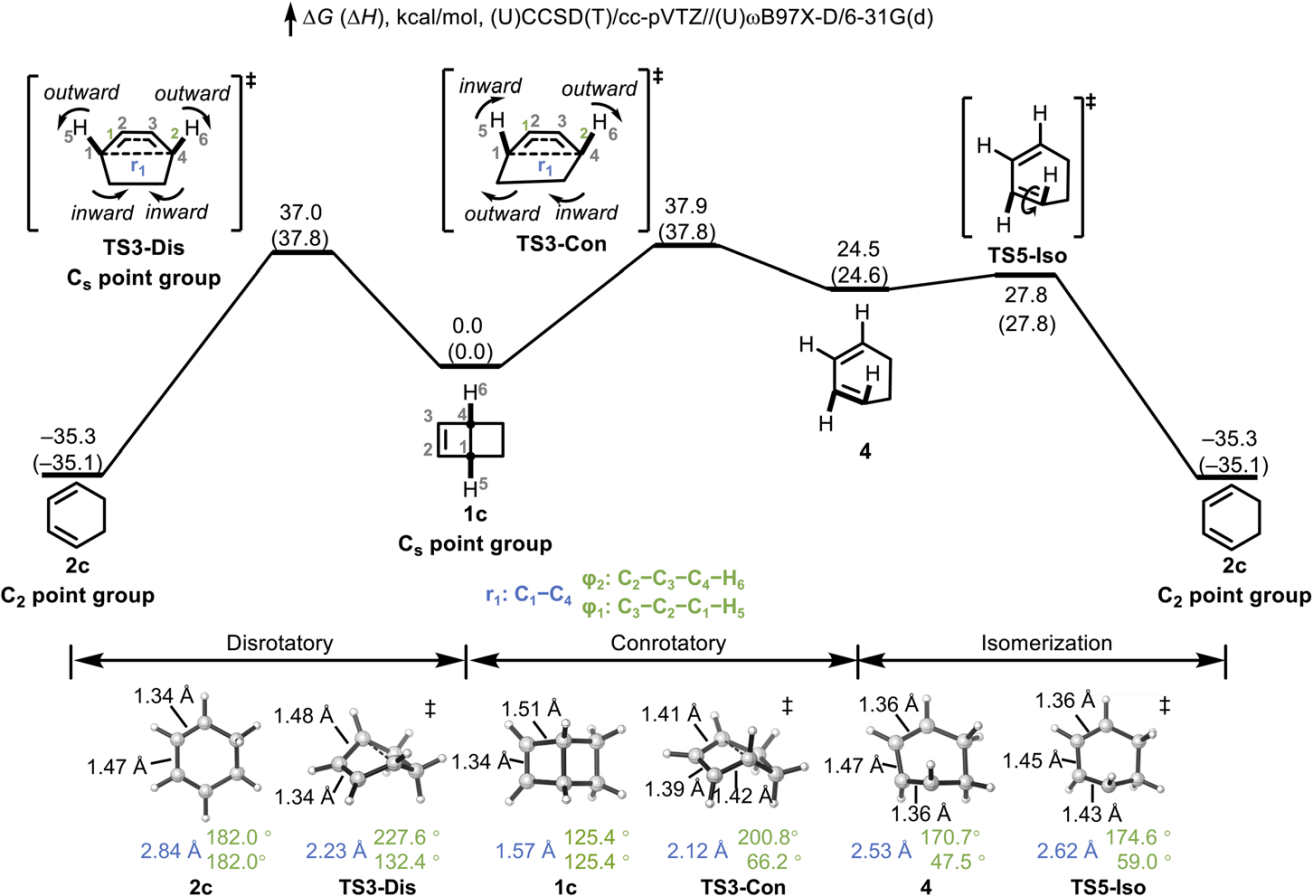
Free energy diagram for the electrocyclic ring opening of bicyclo[2.2.0]hex-2-ene. Energies are in kcal mol^−1^ and are calculated at the (U)CCSD(T)/cc-pVTZ//(U)ωB97X-D/6-31G(d) level of theory.

DFT and CCSD(T) calculations were performed to explore the conrotatory and disrotatory electrocyclic ring opening of bicyclo[2.2.0]hex-2-ene (1c). The computed free energy (Δ*G*) and enthalpy (Δ*H*) profiles are shown in Fig. 1. The disrotatory pathway, which is symmetry-forbidden according to the Woodward–Hoffmann rules, proceeds *via*TS3-Dis to yield the thermodynamically favored *cis*,*cis*-1,3-cyclohexadiene (2c, Δ*G* = −35.3 kcal mol^−1^; Δ*H* = −35.1 kcal mol^−1^). Despite the strong thermodynamic driving force, the associated transition state (TS) is high in energy (Δ*G*^‡^ = 37.0 kcal mol^−1^, Δ*H*^‡^ = 37.8 kcal mol^−1^), reflecting its symmetry-forbidden nature. Notably, the disrotatory motion involves outward twisting of both terminal hydrogens, resulting in relatively smooth geometrical changes without significant steric or angular strain. In contrast, the symmetry-allowed conrotatory pathway proceeds through TS3-Con (Δ*G*^‡^ = 37.9 kcal mol^−1^; Δ*H*^‡^ = 37.8 kcal mol^−1^)—at a barrier height essentially identical to the forbidden pathway—to form the highly strained *cis*,*trans*-diene 4 (Δ*G* = 24.5 kcal mol^−1^; Δ*H* = 24.6 kcal mol^−1^). Although symmetry-allowed, this process is geometrically less favorable due to the requirement of one inward and one outward rotation of the terminal hydrogens, which induces severe distortion of the six-membered ring. The comparable activation energies of TS3-Dis and TS3-Con indicate that both pathways are competitive at elevated temperatures. The highly strained 4 then undergoes rapid isomerization through TS5-Iso (Δ*G*^‡^ = 3.3 kcal mol^−1^; Δ*H*^‡^ = 3.2 kcal mol^−1^), affording the thermodynamically favored product 2c. Regardless of the initial pathway (conrotatory or disrotatory), the final product corresponds to the *cis*,*cis*-isomer 2c.

Such reactions were once discussed by Carpenter as prime examples of the small activation barrier difference between formally allowed and forbidden pathways.^15,48^ In such rare instances, structural factors override the fundamental electronic principles of pericyclic reactions, rendering the barrier difference negligible. However, we note that this apparent energetic similarity masks a substantial electronic preference for the allowed conrotatory process. Specifically, the conrotatory process is 60 kcal mol^−1^ less exergonic than the disrotatory pathway. Based on standard Evans–Polanyi or Marcus theory considerations, the disrotatory path should be favored by approximately half of this thermodynamic difference (∼30 kcal mol^−1^). This thermodynamic advantage essentially offsets the intrinsic orbital symmetry barrier, which is typically estimated at 15–30 kcal mol^−1^ for forbidden electrocyclic reactions. Notably, in the parent cyclobutene system, the disrotatory ring opening pathway is associated with a second-order saddle point (SOSP) with two imaginary frequencies rather than a normal transition state.^49–51^ Recent work by Mirzanejad and Muechler demonstrated that imposing geometric constraints to enforce planarity can eliminate the competing planar-to-gauche rotational imaginary frequency and convert the disrotatory SOSP into a first-order saddle point, thereby enabling selective access to the forbidden product.^52^ In our system, bicyclo[2.2.0]hex-2-ene, the rigid bicyclic scaffold inherently enforces such a constraint, ensuring that the ring-opening proceeds through a single, well-defined disrotatory transition state.

Analysis of the structural parameters in Fig. 1 offers insights into geometric evolution along the reaction coordinate. We highlight three key coordinates that track the bond-breaking and torsional components of the reaction: the C1–C4 bond length (*r*_1_, blue), which monitors the cleavage of the bridging bond, and the C3–C2–C1–H5 and C2–C3–C4–H6 dihedral angles (*φ*_1_ and *φ*_2_, green), which captures the rotatory fashion of electrocyclic process and progress of isomerization. In the starting reactant 1c (*C*_s_ symmetry), the C2=C3 double bond is 1.34 Å, the adjacent C3–C4 bond is 1.51 Å, and the C1–C4 bridging bond is 1.57 Å. The dihedral angle *φ*_1_ and *φ*_2_ are both 125.4°. Along the disrotatory pathway, TS3-Dis shows a partially cleaved C1–C4 bond (2.23 Å), while *φ*_1_ increases modestly to 132.4° and *φ*_2_ to 227.6°, consistent with outward rotation. Notably, the C3–C4 bond shortens to 1.48 Å, suggesting partial π-delocalization in the forming diene system. For the disrotatory product 2c (*C*_2_ symmetry), the C1–C4 bond is fully broken (2.84 Å), and the torsion becomes *φ*_1_ = *φ*_2_ = 182.0°. Concurrently, the C2–C3 bond elongates to 1.47 Å, while C3–C4 shortens to 1.34 Å, indicating the character of a conjugated diene.

Compared to TS3-Dis, the C1–C4 bond is shorter (2.12 *vs.* 2.23 Å) in conrotatory TS3-Con, but the torsional angle *φ*_1_ decreases significantly to 66.2° and *φ*_2_ increases to 200.8°, reflecting the inward rotation characteristic of the conrotatory process. The C2–C3 bond elongates to 1.39 Å, and C3–C4 shortens to 1.41 Å, indicating partial π-bond formation. The conrotatory product 4 (*cis*,*trans*-1,3-cyclohexadiene) exhibits bond lengths characteristic of a normal conjugated diene system (C2–C3 = 1.47 Å and C3–C4 = 1.36 Å), though the geometry remains highly distorted. The isomerization TS5-Iso enables conversion of *cis*,*trans*-1,3-cyclohexadiene 4 to the thermodynamically preferred *cis*,*cis*-1,3-cyclohexadiene 2c. The isomerization *via*TS5-Iso converts the *cis*,*trans*-1,3-cyclohexadiene 4 to the thermodynamically preferred *cis*,*cis*-isomer 2c. Relative to 4, the TS5-Iso exhibits increased dihedral angles, with *φ*_1_ shifting from 47.5° to 59.0° and *φ*_2_ from 170.7° to 174.6°. Concurrently, the C1–C2 bond elongates from 1.36 Å to 1.43 Å, reflecting a transient weakening of π-bond character; this bond subsequently re-contracts to 1.34 Å as the system relaxes into the fully conjugated product 2c.

**Fig. 2:**
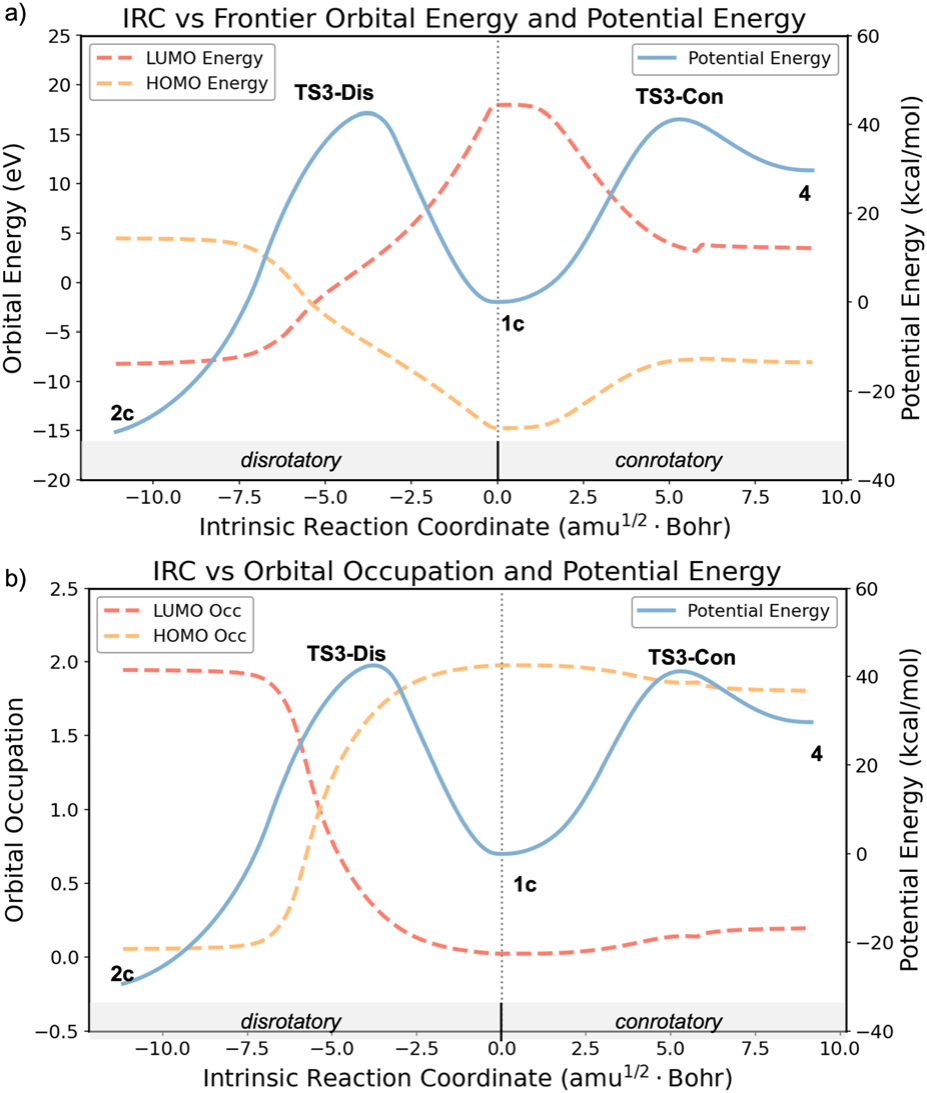
(a) Energies of the frontier molecular orbitals (FMO) and potential energy profiles along the IRCs of the disrotatory and conrotatory ring-opening pathways of bicyclo[2.2.0]hex-2-ene. (b) FMO occupation numbers and potential energy profiles along the same IRCs. Calculations of FMO energies and occupations were performed at the CASSCF(2,2)/def2-SVP level at geometries obtained from IRC calculations with the (U)ωB97X-D/6-31G(d) method.

To gain deeper insights into the electronic structure changes during the ring-opening process, we carried out complete active space self-consistent field (CASSCF) calculations along the intrinsic reaction coordinate (IRC) for both disrotatory and conrotatory trajectories (Fig. 2). These multireference calculations revealed clear distinctions in the frontier molecular orbital (FMO) behavior between the two pathways. As shown in Fig. 2a, the disrotatory pathway is characterized by a crossing of the HOMO and LUMO along the reaction coordinate. Initially, in the reactant (1c), the HOMO and LUMO are energetically well separated. As the system progresses toward TS3-Dis, the energy gap narrows, and the two orbitals cross near the TS3-Dis region. This FMO energetic crossover is also reflected in the orbital occupation numbers plotted in Fig. 2b, where the HOMO occupation gradually decreases from 2.0 to 0.0, while the LUMO occupation increases from 0.0 to 2.0. In the vicinity of TS3-Dis, both orbitals are singly occupied, indicating the formation of a pure diradical.^14^ This continuous shift in occupation from HOMO to LUMO reflects the forbidden and diradical nature of the disrotatory TS. Despite being highly exergonic (Δ*G* = 35.3 kcal mol^−1^, Fig. 1), the pathway is symmetry-forbidden according to the Woodward–Hoffmann rules and displays a significant barrier (∼40 kcal mol^−1^), consistent with the formation of a diradical transition state.

In contrast, there is no such crossing during the conrotatory pathway. As the system follows the IRC through TS3-Con toward the *cis*,*trans*-1,3-cyclohexadiene product (4), the HOMO and LUMO approach each other in energy because of the non-planar distortion of the forming π bond. The orbital occupations remain largely unchanged throughout the process (Fig. 2b), although there is some diradical character developing. The product geometry, remarkably, features a nearly perpendicular C=C–C=C torsion (∼90°), which severely limits π-conjugation and results in significant diradical character. The HOMO and LUMO occupations in 4 are 1.80 and 0.20, respectively, corresponding to 20% diradical character in the highly strained product.^53^ This diradicaloid nature arises from poor overlap between orthogonal π-orbitals in the non-planar structure, rather than from a symmetry-forbidden orbital interaction. The CASSCF calculations reinforce the mechanistic distinction between the two electrocyclic ring-opening pathways. The disrotatory process proceeds through a genuine diradical TS, while leading to favorable thermodynamics. In contrast, the conrotatory pathway exhibits no orbital crossing, but the resulting product is destabilized by large geometric strain and exhibits partial diradical character. The evolution of the frontier molecular orbitals (FMOs) was analyzed at the CASSCF(2,2)/def2-SVP level. Along the conrotatory pathway, the HOMOs and LUMOs of the reactant, transition state, and product are shown in Fig. S1a. The HOMO and LUMO start from σ and σ* orbitals and smoothly transform into the π and π* orbitals of the highly distorted double bond in 4 without any crossing between occupied and unoccupied orbitals. The incorporation of a *trans*-double bond in the six-membered ring causes significant non-planar distortion of the alkene with a CC=CC torsional angle near 90° and pyramidal sp^3^ character of the π and π* orbitals. In contrast, along the disrotatory pathway, the HOMO–LUMO orbital crossing point results in a diradical electronic structure with each orbital singly occupied (Fig. S1b). This multi-reference character is consistent with our broken-symmetry DFT results, which predict substantial spin contamination (〈*S*^2^〉 = 0.74) for the disrotatory transition state (TS3-Dis), comparing to a closed-shell character (〈*S*^2^〉 = 0.00) for the conrotatory transition state (TS3-Con).

**Fig. 3:**
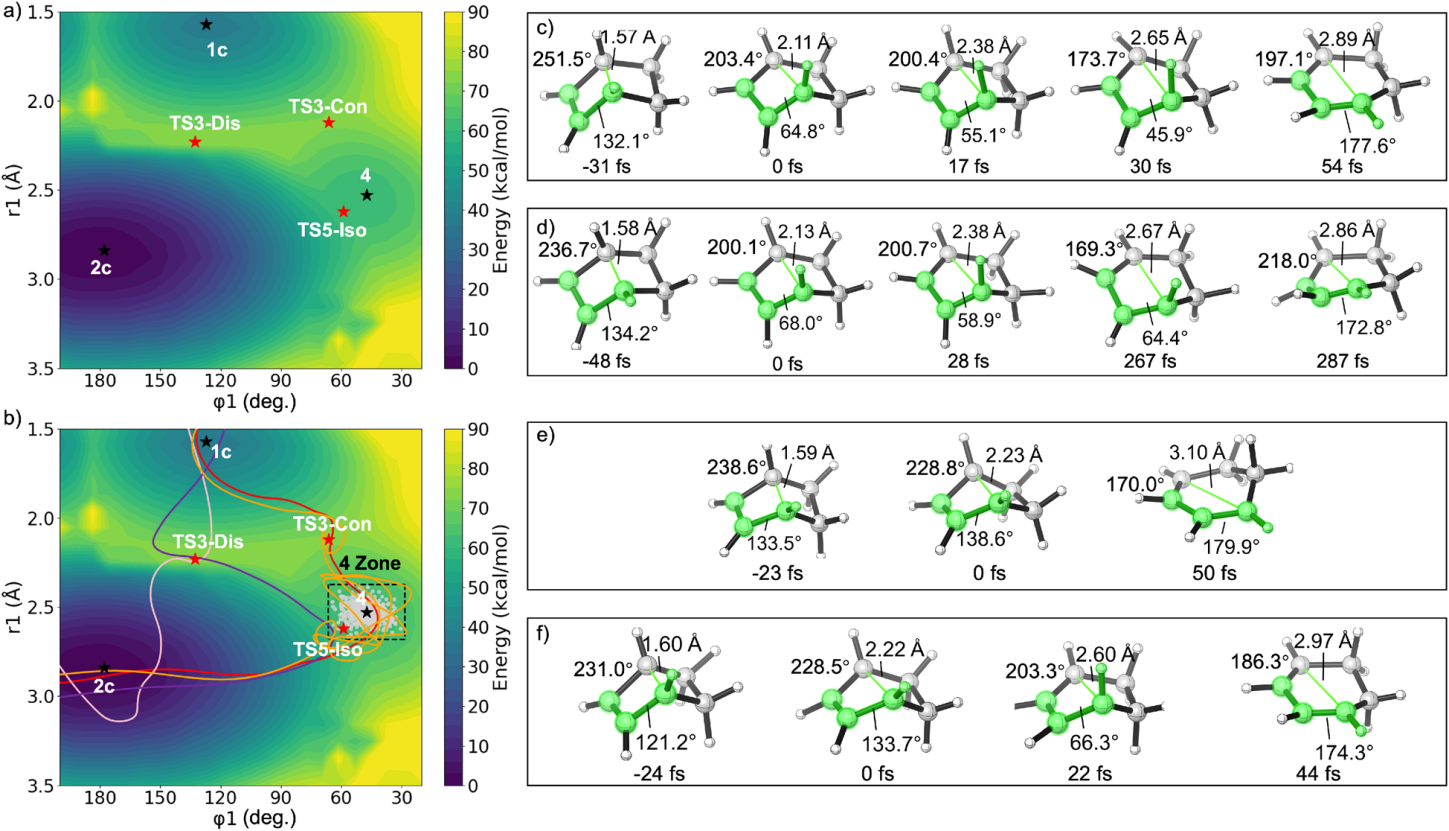
(a) Two-dimensional PES for the electrocyclic ring opening of bicyclo[2.2.0]hex-2-ene, defined by the dihedral angle *φ*_1_ and breaking bond length *r*_1_. 1c is the reactant, 2c is the *cis*,*cis*-product, and 4 is the *cis*,*trans*-intermediate. Black stars represent minima; red stars denote transition states. (b) Four representative trajectories from quasi-classical MD simulations. The gray region (“4 Zone”) encompasses 98% of structures sampled for 4 by normal mode sampling in ProgDyn. Red and orange lines represent conrotatory trajectories (corresponding to snapshots in (c) and (d)); pink and purple lines show disrotatory trajectories ((e) and (f)). All calculations were performed at the (U)ωB97X-D/6-31G(d) level.

We also explored the dynamic behavior of the bicyclo[2.2.0]hex-2-ene electrocyclic reactions. We constructed a two-dimensional potential energy surface (PES) in Fig. 3, using two key reaction coordinates: the breaking bond distance *r*_1_ (C1–C4) and the torsional angle *φ*_1_ (C3–C2–C1–H5). As shown in Fig. 3a, the PES features two distinct saddle points corresponding to the disrotatory (TS3-Dis) and conrotatory (TS3-Con) transition states. The starting material 1c and the two products—*cis*,*trans*-diene 4 and *cis*,*cis*-diene 2c—appear as local minima.

We investigated the dynamic behavior of the system by performing quasi-classical molecular dynamics simulations initiated near the conrotatory and disrotatory TSs using normal mode sampling and propagated in both forward and reverse directions. Representative trajectories are projected onto the same PES in Fig. 3b. In the conrotatory pathway, a total of 226 quasi-classical trajectories (QCTs) were initiated from sampled TS3-Con normal mode points. Among them, 210 trajectories (92.9%) were productive, connecting the reactant 1c and final product 2c, while the other 16 trajectories (7.1%) were recrossing events, returning to the same side of the reaction (1c to 1c or 2c to 2c). All productive conrotatory trajectories projected onto the PES are shown in Fig. S2a. Among them, two conrotatory trajectories are shown in Fig. 3b: a concerted trajectory (red) that proceeds smoothly from TS3-Con to 2c without pausing at 4 (see snapshots in Fig. 3c), and a stepwise trajectory (orange) that temporarily pauses in a region near 4 before continuing to isomerize (Fig. 3d). To statistically quantify the residence time of trajectories in the region of 4, we defined the “4 Zone” as the region in (*r*_1_, *φ*_1_) space that encloses over 98% of geometries from a normal mode sampling ensemble of 4. This region is defined by a bond length (*r*_1_) ranging from 2.37 to 2.68 Å and a dihedral angle (*φ*_1_) ranging from 28.4° and 66.5°, and is shown as the gray shaded area in Fig. 3b.

For the two representative trajectories, the concerted trajectory begins at *t* = 0 fs from a sampled TS3-Con as shown in Fig. 3c. The breaking bond distance *r*_1_ and the torsional angle *φ*_1_ are highlighted in bright green while *φ*_2_ is displayed in the top-left corner of each structure. Backward propagation toward the reactant reaches *r*_1_ < 1.60 Å at *t* = −31 fs, indicating the formation of the reactant. Forward propagation enters the 4 Zone at *t* = 17 fs, with *r*_1_ = 2.38 Å and *φ*_1_ = 55.1°, both within the defined boundaries. The molecule stays in this region for only ∼13 fs (about the period of a C–H vibration) before escaping at *t* = 30 fs. By *t* = 54 fs, the trajectory reaches the final product, characterized by *φ*_1_ ≈ 180°, consistent with the planar diene. *φ*_2_ also exhibits a rapid planarization, decreasing from 251° (at −31 fs) to 173.7° (at 30 fs), indicating the completion of the first electrocyclization. In contrast, the stepwise trajectory in Fig. 3d displays markedly different dynamics. It enters the 4 Zone at *t* = 28 fs (*r*_1_ = 2.38 Å, *φ*_1_ = 58.9°), and then exhibits “jigglings and wigglings”^54^ near the 4 Zone for a longer time. Although thermal fluctuations occasionally bring the structure out of the strict 4 Zone boundaries, it generally remains within this region for over 239 fs, finally exiting the zone at *t* = 267 fs and progressing to the thermodynamic product. *φ*_2_ also gradually approaches planarity, changing from 236.7° (at −48 fs) to 200.1° (at 0 fs), and then to 218.0° (at 287 fs).

In the disrotatory pathway, a total of 195 QCTs were initiated from normal mode sampled TS3-Dis points. Among them, 178 trajectories (91.3%) were productive, while the other 17 trajectories (8.7%) were recrossing. All productive disrotatory trajectories are projected onto the PES in Fig. S2b. Among them, two representative disrotatory trajectories are shown in Fig. 3b. Unlike the conrotatory case, these two disrotatory trajectories (pink and purple) traverse a distinct region of the PES, proceeding directly from TS3-Dis to the *cis*,*cis*-product 2c (Fig. 3e and f). The pink trajectory exhibits a concerted dynamical behavior after TS3-Dis, which can be understood in the context of the relative topography of the potential energy surface: the *cis*,*trans*-diene 4 lies in a shallow energy well as an unstable intermediate, whereas TS3-Dis is a diradical that leads directly to the thermodynamic product 2c.

Because the disrotatory transition state TS3-Dis exhibits significant open-shell (diradical) character (as described in Fig. 2), it is not constrained by orbital symmetry. Trajectories initiated from TS3-Dis explore various directions on the PES. Some trajectories exhibit motion toward the shallow 4 region (purple), as driven by the dynamic momentum. However, due to the thermodynamic stability of 2c, most trajectories lead to 2c without actually visiting 4. Therefore, we observed these two types of concerted disrotatory trajectories from TS3-Dis. As shown in Fig. S2b, for trajectories initiated from TS3-Dis, 87.1% of the trajectories exhibit a direct outward rotation of *φ*_1_, consistent with a classic disrotatory motion, while the remaining 12.9% initially rotate inward in a conrotatory fashion before reversing direction and completing the reaction *via* an overall disrotatory motion.

For the trajectory shown in Fig. 3e, backward propagation from TS3-Dis reaches the reactant 1c (*r*_1_ = 1.59 Å, *φ*_1_ = 133.5°) at *t* = −23 fs. The forward trajectory proceeds with an outward torsional motion, rapidly planarizing to form the *cis*,*cis*-product 2c with *r*_1_ = 3.10 Å and *φ*_1_ = 138.6° at *t* = 50 fs. In contrast, the trajectory shown in Fig. 3f exhibits a different initial behavior. Starting from TS3-Dis, the trajectory undergoes an inward conrotatory rotation, reaching a geometry near the 4 Zone at *t* = 22 fs with *r*_1_ = 2.60 Å and *φ*_1_ = 66.3°. However, it does not remain in this region, but instead rapidly evolves toward the thermodynamically favored *cis*,*cis*-diene product, reaching a near-planar geometry with *r*_1_ = 2.94 Å and *φ*_1_ = 174.3° by *t* = 44 fs.

**Fig. 4:**
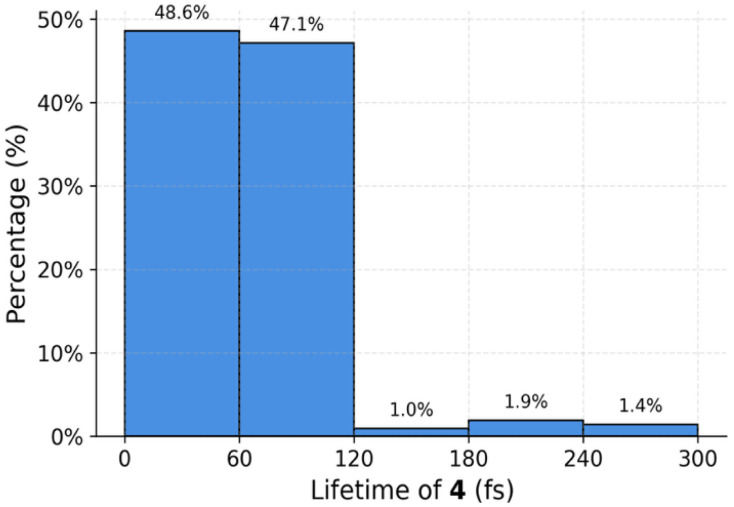
Distribution of the lifetimes of the *cis*,*trans*-1,3-cyclohexadiene 4.

We analyzed the residence time of trajectories within the “4 Zone” defined by *r*_1_ = 2.37–2.68 Å and *φ*_1_ = 28.4–66.5°. Fig. 4 shows the distribution of lifetimes spent within this region across all productive conrotatory trajectories. The lifetime of intermediate 4 is defined as the time interval between the trajectory's first entrance into the “4 Zone” and its exit from this region. The results reveal a highly skewed distribution: the vast majority of trajectories (95.7%; comprising 48.6% < 60 fs and 47.1% < 120 fs) remain in the 4 Zone for less than 120 fs, with only a small fraction (4.3%) persisting for longer than 120 fs. The average lifetime of 4 is calculated to be 56.9 ± 54.2 fs. This behavior stands in sharp contrast to the prediction from transition state theory (TST). The free energy barrier for isomerization from *cis*,*trans*- to *cis*,*cis*-diene is calculated to be 3.3 kcal mol^−1^. According to classical TST, this barrier corresponds to a rate constant (*k*) of 2.37 × 10^−5^ fs^−1^ at 298 K, yielding a predicted half-life (*t*_1/2_) of 2.92 × 10^4^ fs (∼29.2 ps) for 4. While 29.2 ps is short, it is approximately 500 times longer than the dynamically simulated lifetime. The significantly reduced lifetime of 4 highlights its nonstatistical dynamic behavior, driven by dynamic momentum and the absence of rapid intramolecular vibrational redistribution (IVR)^16,28,39,44,55^ during the reaction process.

**Fig. 5:**
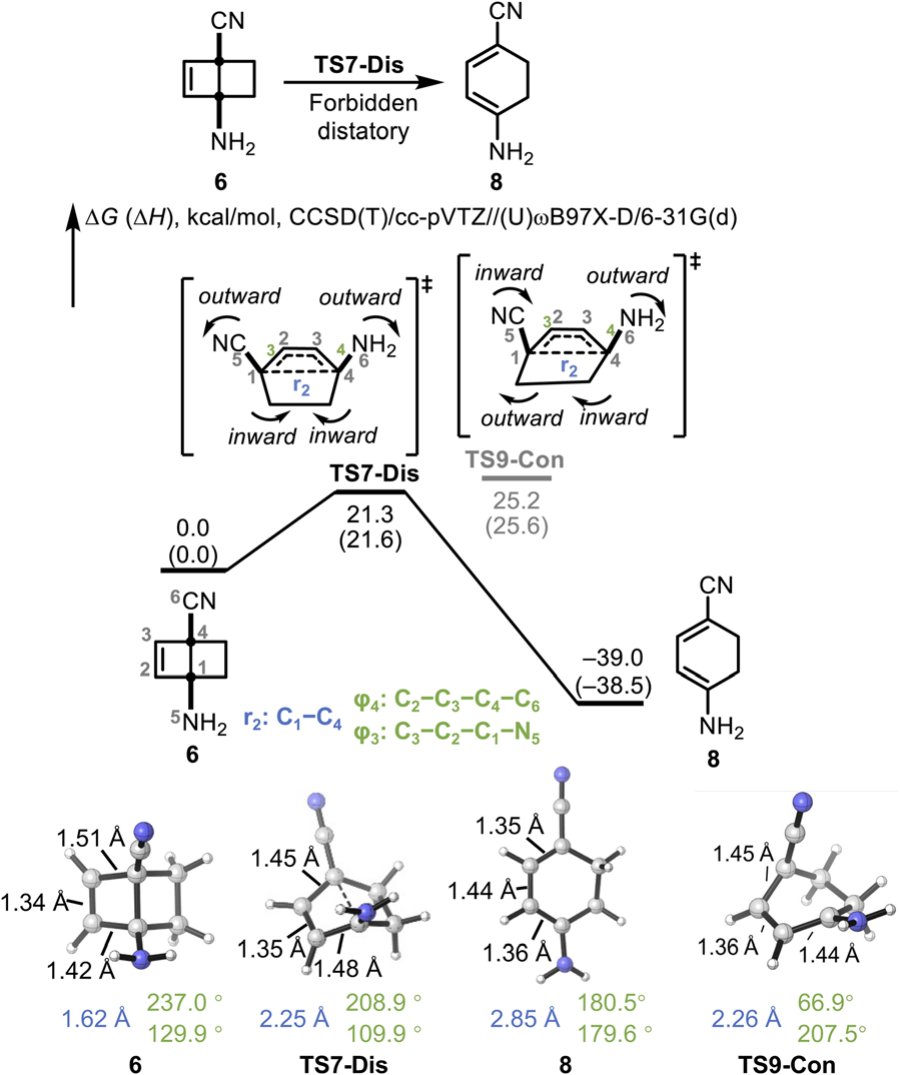
DFT-calculated energy diagram and IRC for the electrocyclic ring opening of 1-amino-4-cyanobicyclo[2.2.0]hex-2-ene. Energies are in kcal mol^−1^ and are calculated at the (U)CCSD(T)/cc-pVTZ//(U)ωB97X-D/6-31G(d) level of theory.

We explored a polarized version of bicyclo[2.2.0]hex-2-ene, with amino and cyano groups at C-1 and C-4 (Fig. 5). We previously showed that donor and acceptor groups at the termini of 6π and 14π electron systems could eliminate any orbital symmetry restrictions.^12,56,57^ DFT and CCSD(T) calculations reveal that the reaction proceeds through a concerted, disrotatory TS7-Dis, leading to the planar diene product (8). Despite the formal Woodward–Hoffmann-forbidden nature of the disrotatory ring opening of a four-electron system, the computed barrier is low (Δ*G*^‡^ = 21.3 kcal mol^−1^, Δ*H*^‡^ = 21.6 kcal mol^−1^), and the reaction is highly exergonic (Δ*G* = −39.0 kcal mol^−1^, Δ*H* = −38.5 kcal mol^−1^). In TS7-Dis, the breaking C1–C4 bond (denoted as *r*_2_) elongates from 1.62 Å in 6 to 2.25 Å. In product 8, the dihedral angle *φ*_3_ (C3–C2–C1–N5) increases from 109.9° (in TS7-Dis) to 179.6° (in 8), and *φ*_4_ (C2–C3–C4–C6) decreases from 208.9° (in TS7-Dis) to 180.5° (in 8), reflecting a clear disrotatory motion. The breaking bond is fully cleaved (2.85 Å in 8) to form a near-planar diene. In addition to the elongation of the breaking *r*_2_ bond, the ring-opening process is accompanied by characteristic changes in the adjacent π- and σ-bonds. In the reactant 6, the C2=C3 double bond length is 1.34 Å, while the neighboring single bonds C1–C2 and C3–C4 are 1.42 Å and 1.51 Å, respectively. At TS7-Dis, these bonds begin to reorganize: the C2=C3 double bond slightly elongates to 1.35 Å, while the former σ-bonds undergo distinct changes—C3–C4 shortens to 1.45 Å, while C1–C2 stretches to 1.48 Å—indicating the onset of π-delocalization across the forming diene system. In the product 8, full π-conjugation is established. The C1=C2 and C3=C4 double bonds are formed with bond lengths of 1.36 Å and 1.35 Å, respectively, while the original double bond becomes a single bond at 1.44 Å. We also located a conrotatory TS9-Con in the polarized system. However, due to the breaking of orbital symmetry constraints caused by charge separation, TS9-Con is not stabilized by closed-shell orbital interactions. In contrast, it lies significantly higher in energy due to the unfavored ring strain (Δ*G*^‡^ = 25.2 kcal mol^−1^). Therefore, TS9-Con will not be visited from 6. Unlike TS3-Con, as shown in Fig. S4, the IRC computed from TS9-Con leads directly to the *cis*,*cis*-product 8, without forming any distinct intermediate.

**Fig. 6:**
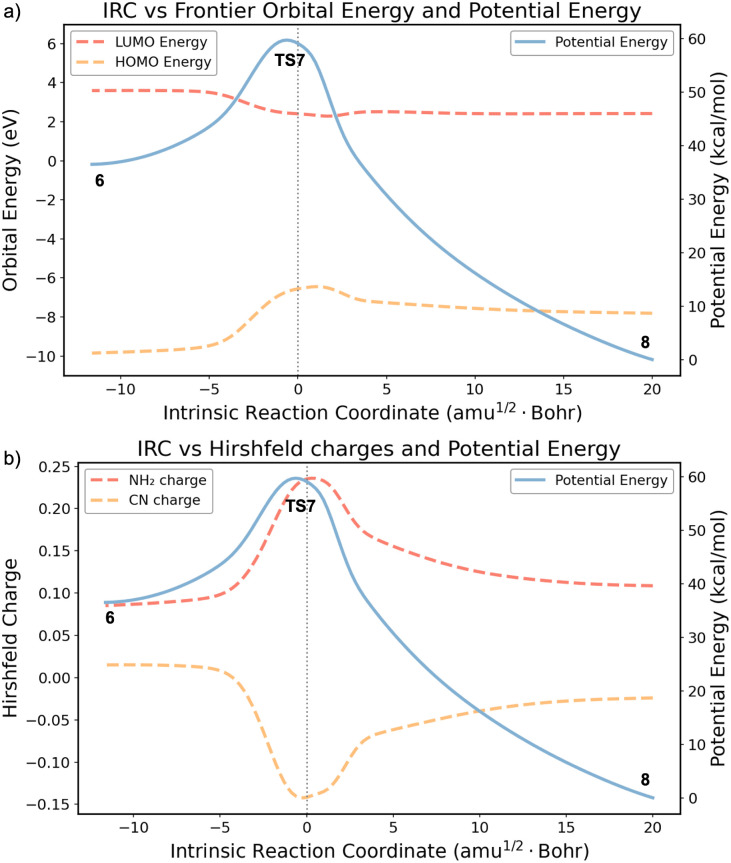
(a) FMO energies along the IRC of the ring-opening process. (b) Calculated CN and NH_2_ termini Hirshfeld charges^58^ along the IRC. Orbital energies and atomic charges were computed using the HF/def2-SVP wavefunction based on ωB97X-D/6-31G(d) optimized geometries.

To further elucidate the origin of such formal violation of the Woodward–Hoffmann rules, we analyzed the evolution of charges and FMO energies along the IRC. As shown in Fig. 6a, this reaction is concerted, specifically, the lowest energy reaction pathway has no intermediate. The IRC exhibits the characteristics of an allowed reaction, with reactant occupied FMOs smoothly transforming into product occupied FMOs. The HOMO and LUMO energies are well-separated throughout the reaction. The absence of HOMO–LUMO crossing indicates that the reaction proceeds without any diradical character which is typically associated with orbital symmetry violations in nonpolar cases.

We further calculated the Hirshfeld charges of the amino (NH_2_) and cyano (CN) termini along the IRC (Fig. 6b). In the reactant region, the NH_2_ moiety carries a partial positive charge, while the CN moiety is slightly negative, consistent with the donor–acceptor character of the substituents. When approaching the TS, the NH_2_ and CN termini charges peak at +0.24 and −0.14, respectively, reflecting enhanced polarization and the formation of a zwitterionic structure. Here, the previously polarized π orbitals become skewed toward the localized orbitals of a carbocation and a carbanion, stabilized by the NH_2_ and CN substituents, respectively. Fig. S3 shows the FMO shapes of the thermal disrotatory pathway from 1-amino-4-cyanobicyclo[2.2.0]hex-2-ene to 1-amino-4-cyano-1,3-cyclohexadiene. This process can be formally viewed as a carbanion attacking a carbocation, which eliminates orbital symmetry restrictions. Additionally, to quantitatively assess the potential contribution of the zwitterionic resonance form [(−)N=C=C⋯C=N(+)H_2_] in the ground state, Mayer Bond Orders (MBOs)^59,60^ were calculated for compound 6*via* Multiwfn.^61,62^ The reliability of the method was confirmed by the reference bonds: the cyano triple bond (C≡N) and the intracyclic double bond (C2=C3) exhibited MBO values of 2.84 and 1.88, respectively, which align well with their formal bond orders. The standard single bonds within the cyclobutene ring (C1–C2 and C3–C4) showed MBO values of 1.00, while the exocyclic C1–N5 bond was 1.07. In contrast, the central C1–C4 bond, which connects the donor and acceptor moieties, displayed an MBO of only 0.88. This value is significantly lower than that of the double bond and even falls below the standard single bond values observed elsewhere in the ring. The absence of elevated bond order for C1–C4 indicates that electron delocalization through the backbone is negligible, suggesting that the zwitterionic resonance structure does not play a significant role in the ground state.

**Fig. 7:**
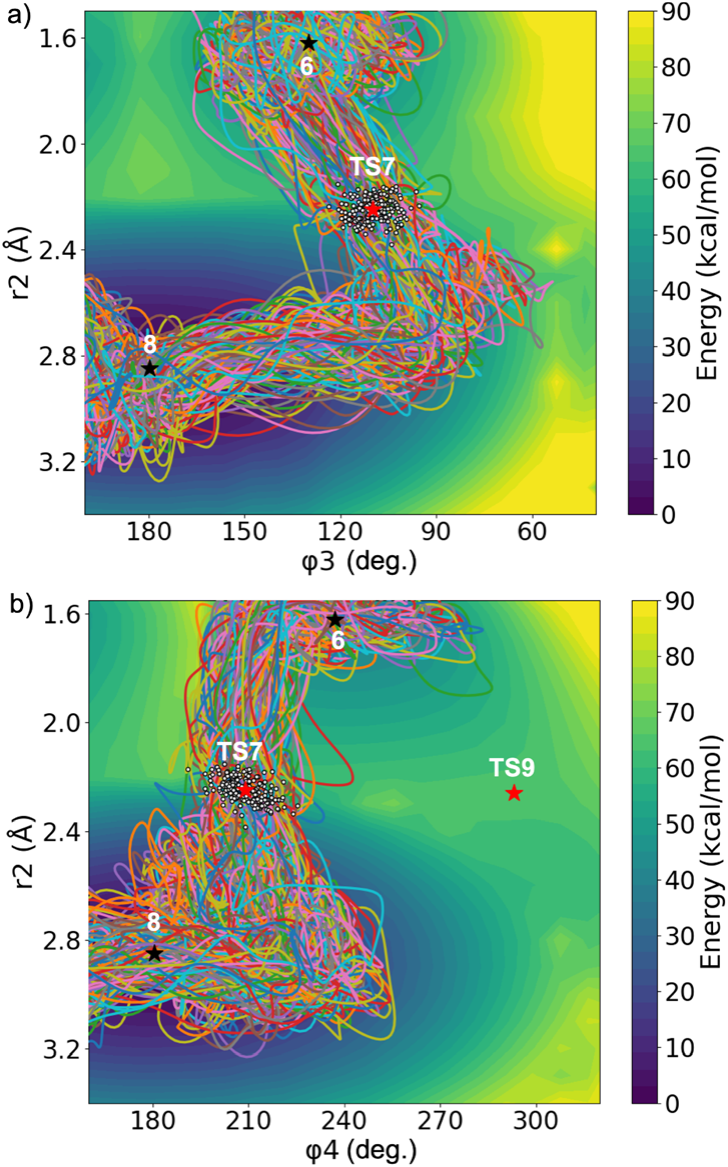
Distributions of all productive trajectories for the disrotatory ring opening initiated from sampled TS7-Dis points. Correlation plots of the breaking bond length *r*_2_*versus* the dihedral angles (a) *φ*_3_; (b) *φ*_4_. Stationary points on the PES are displayed as black stars (minima) and red stars (TSs). The white dots represent the normal mode sampled points where trajectories are initiated. All calculations were performed at the ωB97X-D/6-31G(d) level of theory.

To examine the impact of strong electronic polarization on dynamic behavior, a total of 193 QCTs were initiated from sampled TS7-Dis points. Among them, 181 trajectories (93.8%) were productive, connecting reactant 6 to product 8, while the other 12 trajectories (6.2%) were recrossing. All productive trajectories are shown in Fig. 7. As *r*_2_ elongates, the trajectories first undergo C–C bond cleavage, followed by a rotation of dihedral angles, and finally generate the open-ring product 8. During this process, the C–C bond cleavage and the dihedral angle rotation exhibit an asynchronous mechanism. There is still some semblance of orbital symmetry remaining, as revealed by the trajectories, all of which initially proceed in a disrotatory fashion beyond the transition state, with *φ*_4_ generally decreasing (Fig. 7b) while *φ*_3_ and *r*_2_ increase (Fig. 7a). However, there is no stable *cis*,*trans*-intermediate on this surface, and the zwitterionic structure readily undergoes disrotatory motion to form the final product. This is also corroborated by the PES contour plot in Fig. 7b, where TS9-Con leads to a plateau region on the PES, which readily descends into product 8 without any barrier. Thus, the disrotatory TS is the only feasible one due to the ring strain in the [2.2.0] bicyclic structure.

## Conclusions

We have explored the potential energy surfaces for the electrocyclic ring opening of bicyclo[2.2.0]hex-2-ene and the influence of donor–acceptor substitution. While the allowed conrotatory and forbidden disrotatory ring-opening pathways of the hydrocarbon have similar barriers, there are dramatic differences in the electronic characteristics and energies of the actual processes. The allowed pathway has a relatively high barrier, leading to a very unstable *cis*,*trans*-diene. Our dynamic simulations reveal pronounced non-statistical behavior: this intermediate possesses an ultrashort lifetime, pausing for only a few C–C vibrations before isomerizing to the stable *cis*,*cis*-diene.

The forbidden pathway is driven by relief of strain and has a very similar barrier to the allowed, an example cited by Carpenter in his recent discussion of the meaning, or lack thereof, of allowed and forbidden in this journal.^15^ We note that the concerted but disrotatory forbidden pathway proceeds directly to the very stable *cis*,*cis*-product, but only at the expense of a diradical transition state. Consequently, this pathway is as difficult as the allowed reaction, in spite of its thermodynamic favorability.

We explored the hypothetical 1-amino-4-cyano derivative to test the generality of our discovery that such donor–acceptor substitution can eliminate the W–H rule control of stereochemistry in 14-, 18- and 6-electron electrocyclizations. As we previously noted, Epiotis long ago proposed that donor and acceptor substitution might lead to a preference for formally forbidden pericyclic reactions.^63^ Our previous studies established examples of this in the Prinzbach's 14-, 18- electron systems and several hypothetical 6-electron systems.^12^ We have now shown for a 4-electron system for the first time that strong donor–acceptor substitution can make a geometrically favored forbidden disrotatory electrocyclization favored by transforming the diradical transition state into a relatively stable zwitterionic transition state.

## Author contributions

Z. Q. performed calculations and molecular dynamics simulations. Q. Z. carried out CASSCF calculations and contributed to data analysis, visualization. K. N. H. conceived the project and designed the overall research plan. Z. Q., Q. Z., R.-K. W. and K. N. H. wrote the paper. All authors discussed the results and approved the final version.

## Conflicts of interest

The authors declare no conflicts of interest.

## Supplementary Material

SC-OLF-D5SC07711G-s001

## Data Availability

The data underlying this study are available in the published article and its supplementary information (SI). Supplementary information: computational methods, example inputs for Gaussian and ORCA. Productive trajectories for the ring opening of the parent system. FMO during the ring opening of the parent and polar systems. Conrotatory transition state TS9-Con of the polar system 6. Energies and Cartesian coordinates of all optimized structures. See DOI: https://doi.org/10.1039/d5sc07711g.

## References

[cit1] Goldstein M. J., Leight R. S., Lipton M. S. (1976). J. Am. Chem. Soc..

[cit2] Carpenter B. K. , PATAI's Chemistry of Functional Groups, John Wiley & Sons, Ltd, 2009

[cit3] Durst T. and Breau L., in Comprehensive Organic Synthesis, B. M. Trost and I. Fleming, Pergamon, Oxford, 1991, pp. 675–697

[cit4] Hasselmann D., Loosen K. (1978). Angew Chem. Int. Ed. Engl..

[cit5] Silva López C., Nieto Faza O., de Lera Á. R. (2006). Org. Lett..

[cit6] Silva López C., Nieto Faza O., de Lera Á. R. (2007). Chem.–Eur. J..

[cit7] Baldwin J. E., Gallagher S. S., Leber P. A., Raghavan A. S., Shukla R. (2004). J. Org. Chem..

[cit8] Baldwin J. E., Gallagher S. S., Leber P. A., Raghavan A. (2004). Org. Lett..

[cit9] Woodward R. B., Hoffmann R. (1965). J. Am. Chem. Soc..

[cit10] Woodward R. B., Hoffmann R. (1969). Angew Chem. Int. Ed. Engl..

[cit11] Bajorek T., Werstiuk N. H. (2002). Chem. Commun..

[cit12] Kukier G. A., Turlik A., Xue X.-S., Houk K. N. (2021). J. Am. Chem. Soc..

[cit13] Zhou Q., Kukier G., Gordiy I., Hoffmann R., Seeman J. I., Houk K. N. (2024). J. Org. Chem..

[cit14] Stuyver T., Chen B., Zeng T., Geerlings P., De Proft F., Hoffmann R. (2019). Chem. Rev..

[cit15] Carpenter B. K. (2025). Chem. Sci..

[cit16] Carpenter B. K. (2013). Chem. Rev..

[cit17] Rehbein J., Carpenter B. K. (2011). Phys. Chem. Chem. Phys..

[cit18] Nummela J. A., Carpenter B. K. (2002). J. Am. Chem. Soc..

[cit19] Reyes M. B., Carpenter B. K. (2000). J. Am. Chem. Soc..

[cit20] Carpenter B. K. (1998). Angew. Chem., Int. Ed..

[cit21] Carpenter B. K. (1995). J. Am. Chem. Soc..

[cit22] Carpenter B. K. (1992). Acc. Chem. Res..

[cit23] Newman-Evans R. H., Simon R. J., Carpenter B. K. (1990). J. Org. Chem..

[cit24] Bailey J. O., Singleton D. A. (2017). J. Am. Chem. Soc..

[cit25] Kurouchi H., Andujar-De Sanctis I. L., Singleton D. A. (2016). J. Am. Chem. Soc..

[cit26] Chen Z., Nieves-Quinones Y., Waas J. R., Singleton D. A. (2014). J. Am. Chem. Soc..

[cit27] Biswas B., Collins S. C., Singleton D. A. (2014). J. Am. Chem. Soc..

[cit28] Quijano L. M. M., Singleton D. A. (2011). J. Am. Chem. Soc..

[cit29] Wang Z., Hirschi J. S., Singleton D. A. (2009). Angew. Chem..

[cit30] Oyola Y., Singleton D. A. (2009). J. Am. Chem. Soc..

[cit31] Thomas J. B., Waas J. R., Harmata M., Singleton D. A. (2008). J. Am. Chem. Soc..

[cit32] Bekele T., Christian C. F., Lipton M. A., Singleton D. A. (2005). J. Am. Chem. Soc..

[cit33] Singleton D. A., Hang C., Szymanski M. J., Meyer M. P., Leach A. G., Kuwata K. T., Chen J. S., Greer A., Foote C. S., Houk K. N. (2003). J. Am. Chem. Soc..

[cit34] Törk L., Jiménez-Osés G., Doubleday C., Liu F., Houk K. N. (2015). J. Am. Chem. Soc..

[cit35] Xu L., Doubleday C. E., Houk K. N. (2011). J. Am. Chem. Soc..

[cit36] Doubleday C., Suhrada C. P., Houk K. N. (2006). J. Am. Chem. Soc..

[cit37] Matute R. A., Houk K. N. (2012). Angew. Chem..

[cit38] Jiménez-Osés G., Liu P., Matute R. A., Houk K. N. (2014). Angew. Chem., Int. Ed..

[cit39] Ess D. H. (2021). Acc. Chem. Res..

[cit40] Paul A. K., West N. A., Winner J. D., Bowersox R. D. W., North S. W., Hase W. L. (2018). J. Chem. Phys..

[cit41] Hamaguchi M., Nakaishi M., Nagai T., Nakamura T., Abe M. (2007). J. Am. Chem. Soc..

[cit42] Schmittel M., Vavilala C., Jaquet R. (2007). Angew. Chem..

[cit43] Bach A., Hostettler J. M., Chen P. (2006). J. Chem. Phys..

[cit44] Sun L., Song K., Hase W. L. (2002). Science.

[cit45] Doubleday C., Li G., Hase W. L. (2002). Phys. Chem. Chem. Phys..

[cit46] Rabinovitch B. S., Rynbrandt J. D. (1971). J. Phys. Chem..

[cit47] Reyes M. B., Lobkovsky E. B., Carpenter B. K. (2002). J. Am. Chem. Soc..

[cit48] Carpenter B. K. (2005). Chem. Cyclobutanes.

[cit49] Breulet J., Schaefer H. F. (1984). J. Am. Chem. Soc..

[cit50] Sakai S. (1999). J. Mol. Struct..

[cit51] Brown C. L., Bowser B. H., Meisner J., Kouznetsova T. B., Seritan S., Martinez T. J., Craig S. L. (2021). J. Am. Chem. Soc..

[cit52] Mirzanejad A., Muechler L. (2025). ChemPhysChem.

[cit53] Yamaguchi K. (1975). Chem. Phys. Lett..

[cit54] Feynman R. P. , QED: The Strange Theory of Light and Matter, Princeton University Press, Princeton, NJ, 1985

[cit55] Doubleday C., Boguslav M., Howell C., Korotkin S. D., Shaked D. (2016). J. Am. Chem. Soc..

[cit56] Prinzbach H., Babsch H., Hunkler D. (1978). Tetrahedron Lett..

[cit57] Prinzbach H., Bingmann H., Beck A., Hunkler D., Sauter H., Hädicke E. (1981). Chem. Ber..

[cit58] Hirshfeld F. L. (1977). Theoret. Chim. Acta.

[cit59] Mayer I. (1983). Chem. Phys. Lett..

[cit60] Bridgeman A. J., Cavigliasso G., Ireland L. R., Rothery J. (2001). J. Chem. Soc., Dalton Trans..

[cit61] Lu T. (2024). J. Chem. Phys..

[cit62] Lu T., Chen F. (2012). J. Comput. Chem..

[cit63] Epiotis N. D. (1972). J. Am. Chem. Soc..

