# Supplementary material for: Molecular dynamics of bicyclo[2.2.0]hex-2-ene ring opening and its polar derivative: allowed vs. forbidden pathways
Source: Chem Sci. 2026 Feb 10;17(13):6644–52. doi: 10.1039/d5sc07711g (PMC12888024; doi:10.1039/d5sc07711g)
Supplement: SC-017-D5SC07711G-s001 [file SC-017-D5SC07711G-s001.pdf]

Supplementary Information (SI) for Chemical Science.
This journal is © The Royal Society of Chemistry 2026

*Supporting Information (SI)*

# Molecular Dynamics of Electrocyclic Ring Opening of Bicyclo[2.2.0]hex-2-ene and Its Polar Derivative: Allowed vs. Forbidden Pathways

Zhixin Qin, [†a] Qingyang Zhou, [†b] Rong-Kai Wu [c] and K. N. Houk*[b]

a. SINOPEC (Beijing) Research Institute of Chemical Industry Co. Ltd., Beijing 100013, China.

b. Department of Chemistry and Biochemistry, University of California, Los Angeles, California 90095-1569, United States.

c. Center of Chemistry for Frontier Technologies, Department of Chemistry, Zhejiang University, Hangzhou 310027, China.

† These authors contribute equally to this work.

*E-mail for K. N. Houk: houk@chem.ucla.edu;

**Table of Contents**

I. Computational Details ........ S2
1. Computation Method ........ S2
2. Example Gaussian Input File ........ S2
3. Example ORCA Input File ........ S3
II. <$S^2$> values for all species ........ S3
III. FMO of conrotatory and disrotatory of bicyclo[2.2.0]hex-2-ene ........ S4
IV. Productive trajectories for conrotatory and disrotatory of Bicyclo[2.2.0]hex-2-ene ........ S5
V. FMO of disrotatory of 1-amino-4-cyanobicyclo[2.2.0]hex-2-ene ........ S6
VI. DFT-calculated energy diagram for conrotatory of 1-amino-4-cyanobicyclo[2.2.0]hex-2-ene ... S7
VII. References ........ S7
VIII. Cartesian Coordinates and Energies ........ S9

# I. Computational Details

## 1. Computation Method

All DFT calculations were performed using the Gaussian 16 software package.[1] Geometry optimizations were carried out in the gas phase using the (U)ωB97X-D[2]/6-31G(d) functional. Vibrational frequencies were computed at the same level of theory to evaluate thermodynamic corrections at 298.15 K and 1 atm. Mass-weighted intrinsic reaction coordinate (IRC) [3] calculations were carried out to determine the minimum energy path and ensure that the TSs indeed connect the correct reactants and products. All the potential energy surface contour plots were obtained by scanning breaking and forming bonds at the same level of theory as geometry optimization. Single-point energy calculations were performed at the (U)CCSD(T)[4]/cc-pVTZ[5–9] level on the gas-phase optimized geometries. CASSCF[10]and HF calculations were performed with def2-SVP[11]basis set (with def2-SVP/C[12] and def2/JK[13] as auxiliary basis sets) in ORCA 5.0.[14,15] Plots were generated using in-house Python scripts. The optimized structures for computations were visualized with Legault's CYLview.[16] All the diradical species converge to the stable broken symmetry open-shell singlet wavefunction. The orbitals were visualized using VMD.[17]

Quasi-classical trajectory (QCT) simulations were conducted using Singleton's Progdyn program[18] in conjunction with Gaussian, at the same level of theory as the geometry optimization. To ensure wavefunction stability, the keyword "stable=opt" was used for each trajectory point. Initial conditions for the QCT simulations were sampled from the normal modes of the transition state. Each trajectory was propagated forward and backward for 500 fs using a 1 fs time step with Velocity-Verlet algorithm at 298 K.

## 2. Example Gaussian Input File

```
%nproc=16
%mem=16GB
#p uwb97xd/6-31g* opt=(calcfc,ts,noeigen) freq guess=mix

title

0 1
C         0.564355    0.087205     0.784094
C         0.564355    0.087205    -0.784094
```

```
C       -0.262802 -1.234646      0.774903
C       -0.262802 -1.234646     -0.774903
C       -0.262802  1.345864      0.669761
C       -0.262802  1.345864     -0.669761
H       -1.239693 -1.173734      1.261988
H        0.284204 -2.080907      1.199267
H        0.284204 -2.080907     -1.199267
H       -1.239693 -1.173734     -1.261988
H       -0.760089  1.958298      1.417390
H       -0.760089  1.958298     -1.417390
H        1.483079  0.105807     -1.374228
H        1.483079  0.105807      1.374228
```

### 3. Example ORCA Input File

```
! def2-SVP def2-SVP/C def2/JK miniprint
%maxcore 1000
%pal nprocs 12 end
%casscf
trafostep RI
nel 4
norb 4
mult 1
nroots 1
end
* xyz 0 1
C        0.564355  0.087205      0.784094
C        0.564355  0.087205     -0.784094
C       -0.262802 -1.234646      0.774903
C       -0.262802 -1.234646     -0.774903
C       -0.262802  1.345864      0.669761
C       -0.262802  1.345864     -0.669761
H       -1.239693 -1.173734      1.261988
H        0.284204 -2.080907      1.199267
H        0.284204 -2.080907     -1.199267
H       -1.239693 -1.173734     -1.261988
H       -0.760089  1.958298      1.417390
H       -0.760089  1.958298     -1.417390
H        1.483079  0.105807     -1.374228
H        1.483079  0.105807      1.374228
*
```

## II. $\langle S^2 \rangle$ values for all species

**Table S1.** Calculated <$S^2$> values for all species at (U)CCSD(T) /cc-pVTZ level of theory.

| Structure | <$S^2$> |
|---|---|
| **1c** | 0.0000 |
| **TS3-Con** | 0.0000 |
| **TS3-Dis** | 0.7431 |
| **4** | 0.0000 |
| **TS5-Iso** | 0.6926 |
| **2c** | 0.0000 |
| **6** | 0.0000 |
| **TS7-Dis** | 0.0000 |
| **8** | 0.0000 |
| **TS9-Con** | 0.0000 |

# III. FMO of conrotatory and disrotatory of bicyclo[2.2.0]hex-2-ene

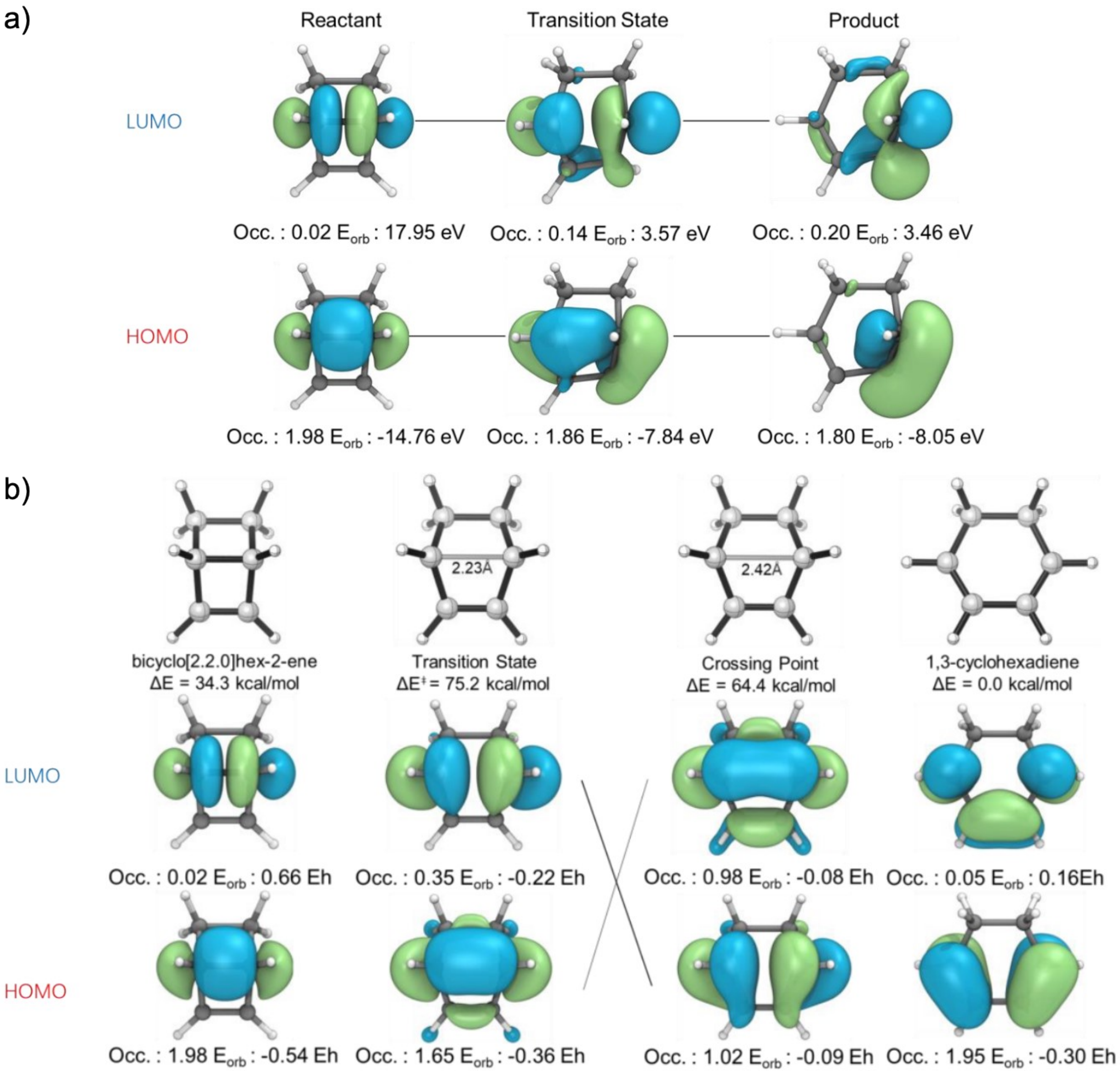


**Fig. S1.** Evolution of the natural orbitals included in the active space for CASSCF(2,2) calculations. a) Orbitals corresponding to the HOMO and LUMO of the reactant, transition state, and product along the conrotatory pathway. b) Orbitals of the reactant, transition state, crossing point, and product along the disrotatory pathway. Note that at the crossing point, the HOMO and LUMO become nearly degenerate (occupancy ~1.0).

# IV. Productive trajectories for conrotatory and disrotatory of bicyclo[2.2.0]hex-2-ene

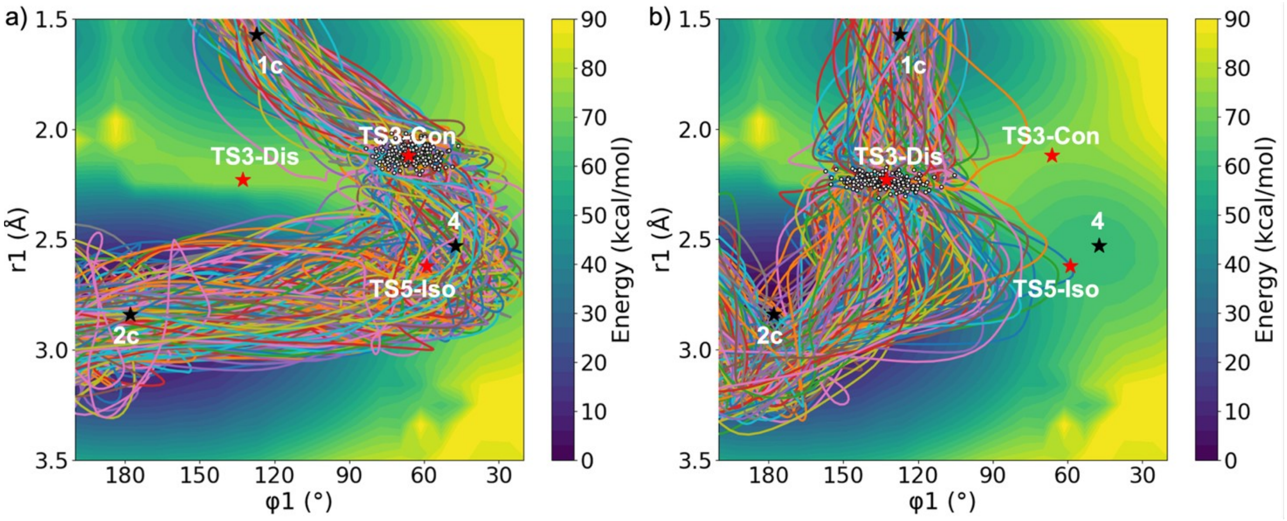


**Fig. S2.** Distributions of all productive trajectories for a) conrotatory opening from sampled **TS3-Con** and b) disrotatory opening from sampled **TS3-Dis** points. Stationary points in the PES are displayed as black stars (minimum) and red stars (TS). The X axis is the dihedral angle of $\varphi_1$, and the Y axis is the distances of breaking bond $r_1$. The white dots are the normal mode sampled points where trajectories are initiated. All calculations were performed at the (U)ωB97X-D/6-31G(d) level of theory.

# V. FMO of disrotatory of 1-amino-4-cyanobicyclo[2.2.0]hex-2-ene

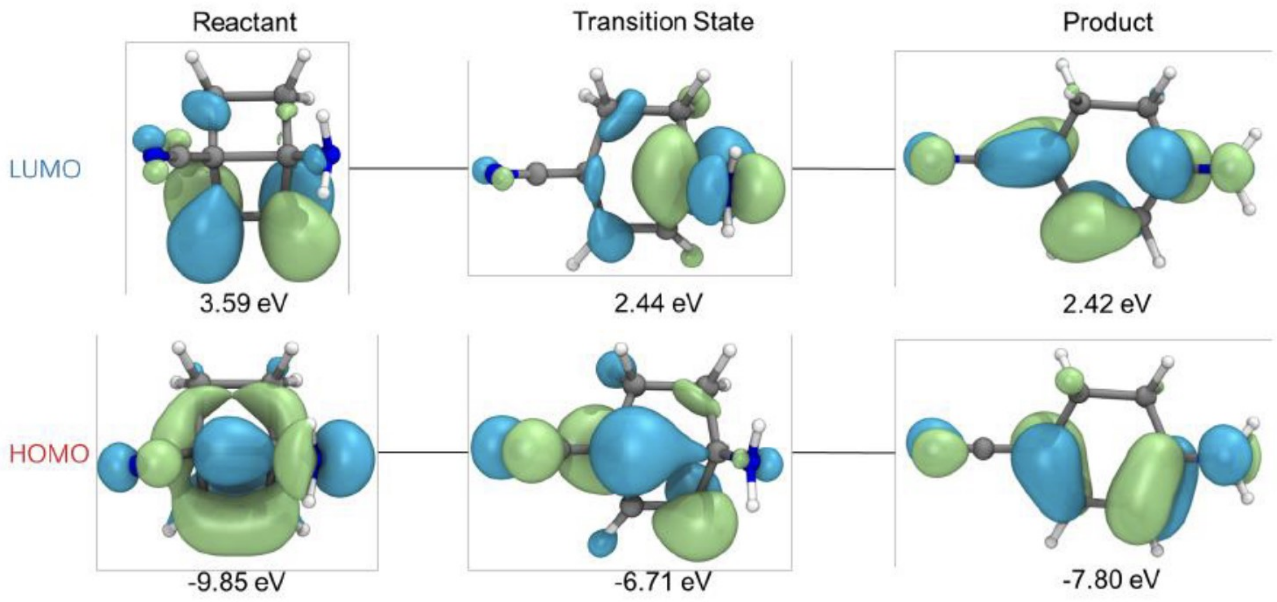


**Fig. S3.** FMO of the thermal disrotatory from 1-amino-4-cyanobicyclo[2.2.0]hex-2-ene to 1-amino-4-cyano-1,3cyclohexadiene.

**Fig. S3** shows the FMOs of reactant, transition state, and product, the highlight of which is the localization observed in the HOMO and LUMO of the transition state. This reaction is concerted,

that is, no intermediates are involved. The IRC has the characteristics of an allowed reaction, with reactant occupied MOs smoothly transforming into product occupied MOs. As the double bonds are broken, the polarized bonding π orbitals become more like the localized orbitals of a carbocation and a carbanion, and the previously delocalized charges located on the CN and $NH_2$ termini concentrate, causing a significant increase in charge separation. Because of the significant polar asymmetry involved, all of these FMOs are asymmetric.

# VI. DFT-calculated energy diagram for conrotatory of 1-amino-4-cyanobicyclo[2.2.0]hex-2-ene

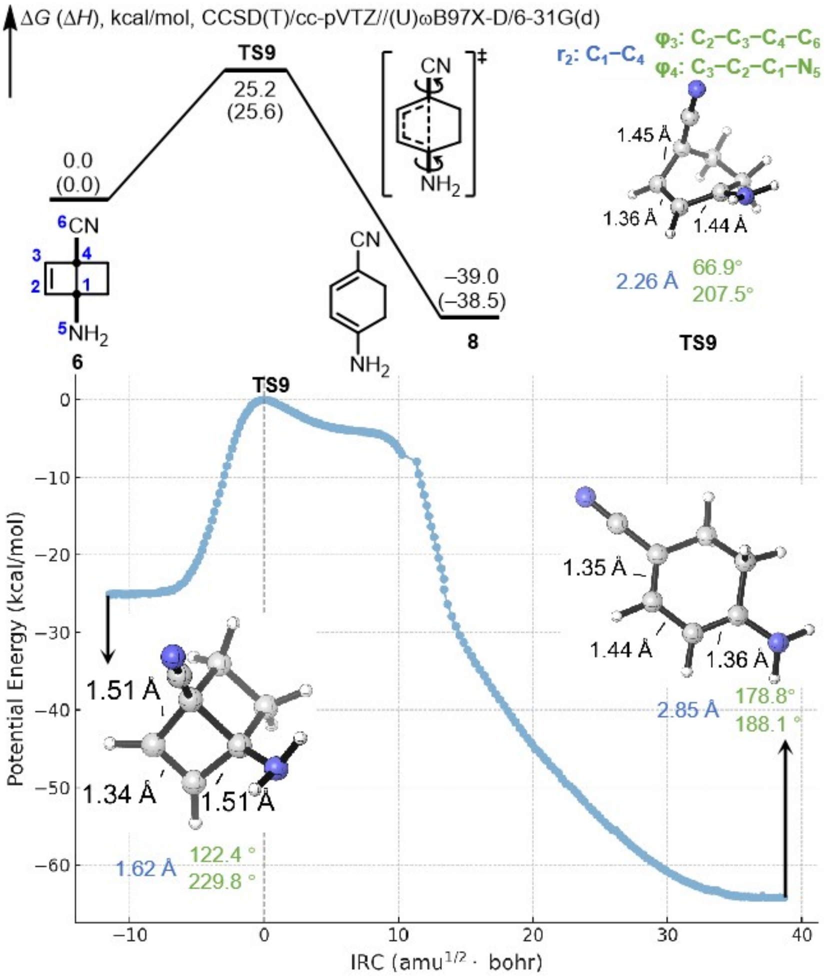


**Fig. S4.** DFT-calculated energy diagram and IRC for the electrocyclic conrotatory ring opening of 1-amino-4-cyanobicyclo[2.2.0]hex-2-ene. Energies are in kcal/mol and are calculated at the (U)CCSD(T)/cc-pVTZ//(U)ωB97X-D/6-31G(d) level of theory.

## VII. References

(1) Gaussian 16, Revision C. 01, Frisch, M. J.; Trucks, G. W.; Schlegel, H. B.; Scuseria, G. E.; Robb, M. A.; Cheeseman, J. R.; Scalmani, G.; Barone, V.; Petersson, G. A.; Nakatsuji, H.; Li, X.; Caricato, M.; Marenich, A. V.; Bloino, J.; Janesko, B. G.; Gomperts, R.; Men-nucci, B.; Hratchian, H. P.; Ortiz, J. V.; Izmaylov, A. F.; Sonnen-berg, J. L.; Williams-Young, D.; Ding, F.; Lipparini, F.; Egidi, F.; Goings, J.; Peng, B.; Petrone, A.; Henderson, T.; Ranasinghe, D.; Zakrzewski, V. G.; Gao, J.; Rega, N.; Zheng, G.; Liang, W.; Hada, M.; Ehara, M.; Toyota, K.; Fukuda, R.; Hasegawa, J.; Ishida, M.; Nakajima, T.; Honda, Y.; Kitao, O.; Nakai, H.; Vreven, T.; Thros-sell, K.; Montgomery, J. A. Jr.; Peralta, J. E.; Ogliaro, F.; Bearpark, M. J.; Heyd, J. J.; Brothers, E. N.; Kudin, K. N.; Staroverov, V. N.; Keith, T. A.; Kobayashi, R.; Normand, J.; Raghavachari, K.; Rendell, A. P.; Burant, J. C.; Iyengar, S. S.; Tomasi, J.; Cossi, M.; Millam, J. M.; Klene, M.; Adamo, C.; Cammi, R.; Ochterski, J. W.; Martin, R. L.; Morokuma, K.; Farkas, O.; Foresman, J. B.; Fox, D. J., Gaussian, Inc., Wallingford CT, 2016.

(2) Chai, J.-D.; Head-Gordon, M. Long-Range Corrected Hybrid Density Functionals with Damped Atom–Atom Dispersion Corrections. Phys. Chem. Chem. Phys. **2008**, 10 (44), 6615–6620. https://doi.org/10.1039/B810189B.

(3) Fukui, K. The Path of Chemical Reactions - the IRC Approach. Acc. Chem. Res. **1981**, 14 (12), 363–368. https://doi.org/10.1021/ar00072a001.

(4) Raghavachari, K. Historical Perspective on: A Fifth-Order Perturbation Comparison of Electron Correlation Theories [Volume 157, Issue 6, 26 May 1989, Pages 479–483]. Chemical Physics Letters **2013**, 589, 35–36. https://doi.org/10.1016/j.cplett.2013.08.044.

(5) Dunning, T. H., Jr. Gaussian Basis Sets for Use in Correlated Molecular Calculations. I. The Atoms Boron through Neon and Hydrogen. The Journal of Chemical Physics **1989**, 90 (2), 1007–1023. https://doi.org/10.1063/1.456153.

(6) Kendall, R. A.; Dunning, T. H., Jr.; Harrison, R. J. Electron Affinities of the First-row Atoms Revisited. Systematic Basis Sets and Wave Functions. The Journal of Chemical Physics **1992**, 96 (9), 6796–6806. https://doi.org/10.1063/1.462569.

(7) Woon, D. E.; Dunning, T. H., Jr. Gaussian Basis Sets for Use in Correlated Molecular Calculations. III. The Atoms Aluminum through Argon. The Journal of Chemical Physics **1993**, 98 (2), 1358–1371. https://doi.org/10.1063/1.464303.

(8) Peterson, K. A.; Woon, D. E.; Dunning, T. H., Jr. Benchmark Calculations with Correlated Molecular Wave Functions. IV. The Classical Barrier Height of the H+H2→H2+H Reaction. The Journal of Chemical Physics **1994**, 100 (10), 7410–7415. https://doi.org/10.1063/1.466884.

(9) Wilson, A. K.; van Mourik, T.; Dunning, T. H. Gaussian Basis Sets for Use in Correlated Molecular Calculations. VI. Sextuple Zeta Correlation Consistent Basis Sets for Boron through Neon. Journal of Molecular Structure: THEOCHEM **1996**, 388, 339–349. https://doi.org/10.1016/S0166-1280(96)80048-0.

(10) Taylor, P. R.; Siegbahn, P. E. M. A COMPLETE ACTIVE SPACE'S& METHOD- (CA&F) U&G A DEiiiITY MATRM. : FORMULATED SUPER-CT APPROACH : Chemical Physics **1980**, 48, 157–173.

(11) Weigend, F.; Ahlrichs, R. Balanced Basis Sets of Split Valence, Triple Zeta Valence and Quadruple Zeta Valence Quality for H to Rn: Design and Assessment of Accuracy. Phys. Chem. Chem. Phys. **2005**, 7 (18), 3297. https://doi.org/10.1039/b508541a.

(12) Hellweg, A.; Hättig, C.; Höfener, S.; Klopper, W. Optimized Accurate Auxiliary Basis Sets for RI-MP2 and RI-CC2 Calculations for the Atoms Rb to Rn. Theor Chem Acc **2007**, 117 (4), 587–597. https://doi.org/10.1007/s00214-007-0250-5.

(13) Weigend, F. Hartree–Fock Exchange Fitting Basis Sets for H to Rn †. J Comput Chem **2008**, 29 (2), 167–175. https://doi.org/10.1002/jcc.20702.

(14) Neese, F. Software Update: The ORCA Program System, Version 4.0. WIREs Comput Mol Sci **2018**, 8 (1). https://doi.org/10.1002/wcms.1327.
(15) Neese, F. The ORCA Program System. WIREs Comput Mol Sci **2012**, 2 (1), 73–78. https://doi.org/10.1002/wcms.81.
(16) Legault, C. Y. CYLview, 1.0b; Université de Sherbrooke: Canada, 2009. http://www.cylview.org.
(17) Humphrey, W.; Dalke, A.; Schulten, K. VMD: Visual Molecular Dynamics. Journal of Molecular Graphics **1996**, 14 (1), 33–38. https://doi.org/10.1016/0263-7855(96)00018-5.
(18) Ussing, B. R.; Hang, C.; Singleton, D. A. Dynamic Effects on the Periselectivity, Rate, Isotope Effects, and Mechanism of Cycloadditions of Ketenes with Cyclopentadiene. J. Am. Chem. Soc. **2006**, 128 (23), 7594–7607. https://doi.org/10.1021/ja0606024.

## VIII. Cartesian Coordinates and Energies

**1c**

E=-233.288289
E_SP=-232.920402
H=-232.790997
G=-232.824853
Imag. Freq. 0

Cartesian coordinates

| | | | |
|---|---|---|---|
| C | 0.564355 | 0.087205 | 0.784094 |
| C | 0.564355 | 0.087205 | -0.784094 |
| C | -0.262802 | -1.234646 | 0.774903 |
| C | -0.262802 | -1.234646 | -0.774903 |
| C | -0.262802 | 1.345864 | 0.669761 |
| C | -0.262802 | 1.345864 | -0.669761 |
| H | -1.239693 | -1.173734 | 1.261988 |
| H | 0.284204 | -2.080907 | 1.199267 |
| H | 0.284204 | -2.080907 | -1.199267 |
| H | -1.239693 | -1.173734 | -1.261988 |
| H | -0.760089 | 1.958298 | 1.417390 |
| H | -0.760089 | 1.958298 | -1.417390 |
| H | 1.483079 | 0.105807 | -1.374228 |
| H | 1.483079 | 0.105807 | 1.374228 |

**2c**

E=-233.34019
E_SP=-232.977134
H=-232.846893
G=-232.880535
Imag. Freq. 0

Cartesian coordinates

| | | | |
|---|---|---|---|
| C | -1.420012 | -0.066057 | 0.112111 |
| C | 1.420012 | 0.066057 | 0.112111 |
| C | -0.725663 | 0.247698 | -1.190680 |
| C | 0.725663 | -0.247698 | -1.190680 |
| C | -0.725663 | -0.109472 | 1.255483 |
| C | 0.725663 | 0.109472 | 1.255483 |
| H | -0.742196 | 1.339328 | -1.339374 |
| H | -1.269326 | -0.186724 | -2.036123 |
| H | 0.742196 | -1.339328 | -1.339374 |
| H | 1.269326 | 0.186724 | -2.036123 |
| H | -1.225929 | -0.289234 | 2.203381 |
| H | 1.225929 | 0.289234 | 2.203381 |
| H | 2.499250 | 0.195389 | 0.110635 |
| H | -2.499250 | -0.195389 | 0.110635 |

**4**

E=-233.24095
E_SP=-232.88047
H=-232.751724
G=-232.785834
Imag. Freq. 0

Cartesian coordinates

| | | | |
|---|---|---|---|
| C | 0.392962 | -1.103228 | 0.496222 |
| C | -0.725482 | 1.115591 | 0.038445 |
| C | 1.474582 | -0.343283 | -0.189980 |
| C | 0.803829 | 1.103761 | -0.043210 |
| C | -0.757664 | -1.189965 | -0.223861 |
| C | -1.542629 | 0.032837 | 0.024295 |
| H | 1.564926 | -0.600106 | -1.250355 |
| H | 2.467541 | -0.346415 | 0.271993 |
| H | 1.187909 | 1.536771 | 0.889327 |
| H | 1.131145 | 1.779746 | -0.846060 |
| H | -0.709355 | -1.514723 | -1.266599 |
| H | -2.626085 | 0.139769 | -0.008791 |
| H | -1.166067 | 2.113819 | 0.057929 |
| H | 0.276399 | -0.803143 | 1.541087 |

**6**

E=-380.837824
E_SP=-380.297057
H=-380.148699
G=-380.18979

Imag. Freq. 0

Cartesian coordinates

| | | | |
|---|---|---|---|
| C | 0.819635 | 0.490428 | -0.058290 |
| C | -0.451178 | -0.480985 | 0.173151 |
| C | 1.344218 | -0.509799 | -1.124526 |
| C | 0.138563 | -1.452872 | -0.895388 |
| C | 1.179514 | 0.130459 | 1.363879 |
| C | 0.122677 | -0.663491 | 1.561813 |
| H | 2.338539 | -0.917513 | -0.928425 |
| H | 1.343741 | -0.065463 | -2.124523 |
| H | -0.503246 | -1.556041 | -1.772679 |
| H | 0.368941 | -2.446772 | -0.506149 |
| H | 2.061404 | 0.365449 | 1.952544 |
| H | -0.181089 | -1.324361 | 2.366975 |
| C | -1.794411 | 0.014757 | 0.002564 |
| N | -2.847894 | 0.470595 | -0.164703 |
| N | 0.809687 | 1.863252 | -0.418235 |
| H | 0.320477 | 2.424651 | 0.272327 |
| H | 0.364572 | 2.012126 | -1.318721 |

**8**

E=-380.900283
E_SP=-380.359852
H=-380.210114
G=-380.251883
Imag. Freq. 0

Cartesian coordinates

| | | | |
|---|---|---|---|
| C | -1.721546 | 0.108675 | -0.023087 |
| C | 1.129770 | 0.072783 | 0.052032 |
| C | -1.004745 | -1.195729 | -0.286295 |
| C | 0.391649 | -1.218830 | 0.336950 |
| C | -1.008866 | 1.259449 | 0.073165 |
| C | 0.431113 | 1.227789 | -0.027185 |
| H | -0.930067 | -1.320973 | -1.376929 |
| H | -1.594273 | -2.036375 | 0.095486 |
| H | 0.304764 | -1.342271 | 1.426531 |
| H | 0.950457 | -2.080617 | -0.037856 |
| H | -1.510797 | 2.216862 | 0.176571 |
| H | 0.959156 | 2.166970 | -0.165206 |
| C | 2.551763 | 0.058148 | -0.037846 |
| N | 3.710874 | 0.015903 | -0.110703 |
| N | -3.087709 | 0.039955 | 0.067657 |
| H | -3.610281 | 0.899454 | -0.017557 |
| H | -3.545942 | -0.767758 | -0.326114 |

**TS3-Con**

E=-233.22259
E_SP=-232.857475
H=-232.730687
G=-232.764432
Imag. Freq. -581.84

Cartesian coordinates

| | | | |
|---|---|---|---|
| C | 0.121581 | -0.900721 | 0.587243 |
| C | -0.410519 | 1.114186 | 0.216992 |
| C | 1.395299 | -0.636035 | -0.151724 |
| C | 1.065292 | 0.888418 | -0.240700 |
| C | -1.065467 | -0.993421 | -0.179928 |
| C | -1.515154 | 0.324871 | -0.147228 |
| H | 1.401326 | -1.116096 | -1.134216 |
| H | 2.332739 | -0.844099 | 0.371465 |
| H | 1.697775 | 1.396566 | 0.493710 |
| H | 1.266833 | 1.353630 | -1.217298 |
| H | -1.244841 | -1.739151 | -0.950700 |
| H | -2.512261 | 0.718135 | -0.356445 |
| H | -0.571663 | 2.112345 | 0.637359 |
| H | 0.083902 | -0.665113 | 1.648199 |

**TS3-Dis**

E=-233.220456
E_SP=-232.855668
H=-232.730806
G=-232.765918
Imag. Freq. -717.46

Cartesian coordinates

| | | | |
|---|---|---|---|
| C | 0.474057 | 0.091421 | 1.114477 |
| C | 0.474057 | 0.091421 | -1.114477 |
| C | -0.212550 | -1.222472 | 0.772130 |
| C | -0.212550 | -1.222472 | -0.772130 |
| C | -0.212550 | 1.328236 | 0.667951 |
| C | -0.212550 | 1.328236 | -0.667951 |
| H | -1.218408 | -1.291007 | 1.210755 |
| H | 0.367589 | -2.065507 | 1.158819 |
| H | 0.367589 | -2.065507 | -1.158819 |
| H | -1.218408 | -1.291007 | -1.210755 |
| H | -0.686444 | 2.052171 | 1.329660 |
| H | -0.686444 | 2.052171 | -1.329660 |
| H | 1.243516 | 0.121233 | -1.882923 |

H 1.243516 0.121233 1.882923

**TS5-Iso**

E=-233.238751
E_SP=-232.872651
H=-232.746684
G=-232.780628
Imag. Freq. -340.95

Cartesian coordinates

| | | | |
|---|---|---|---|
| C | 0.323148 | -1.211721 | 0.473401 |
| C | -0.560793 | 1.223787 | 0.062260 |
| C | 1.379499 | -0.464627 | -0.281064 |
| C | 0.943129 | 1.023511 | 0.002215 |
| C | -0.959805 | -1.107415 | -0.145288 |
| C | -1.489083 | 0.231984 | -0.008171 |
| H | 1.332332 | -0.666711 | -1.356852 |
| H | 2.406801 | -0.623158 | 0.063711 |
| H | 1.378166 | 1.310887 | 0.968755 |
| H | 1.371684 | 1.714154 | -0.737770 |
| H | -1.199371 | -1.671597 | -1.048352 |
| H | -2.543958 | 0.476268 | -0.127830 |
| H | -0.894878 | 2.260565 | 0.076409 |
| H | 0.332656 | -0.973520 | 1.541808 |

**TS7**

E=-380.800779
E_SP=-380.260902
H=-380.114325
G=-380.155815
Imag. Freq. -385.05

Cartesian coordinates

| | | | |
|---|---|---|---|
| C | 1.389189 | -0.095370 | 0.198029 |
| C | -0.809539 | -0.030088 | -0.259237 |
| C | 1.298706 | 1.304020 | -0.332298 |
| C | -0.221682 | 1.349918 | -0.619030 |
| C | 1.115062 | -1.220713 | -0.727633 |
| C | -0.233844 | -1.214078 | -0.855273 |
| H | 1.908421 | 1.384038 | -1.239196 |
| H | 1.634906 | 2.065106 | 0.381426 |
| H | -0.679636 | 2.096502 | 0.034106 |
| H | -0.430816 | 1.651307 | -1.654977 |
| H | 1.875584 | -1.695048 | -1.340355 |
| H | -0.813847 | -1.995699 | -1.350290 |

| | | | |
|---|---|---|---|
| C | -2.117007 | -0.054812 | 0.273189 |
| N | -3.184522 | -0.069639 | 0.746275 |
| N | 1.809746 | -0.337368 | 1.427291 |
| H | 1.772191 | -1.289819 | 1.767529 |
| H | 1.831322 | 0.399392 | 2.120315 |

**TS9**

E=-380.797872
E_SP=-380.254174
H=-380.10789
G=-380.149676
Imag. Freq. -299.37

| | | | |
|---|---|---|---|
| C | -1.07981500 | 0.16487200 | 0.00446800 |
| C | 1.03707000 | -0.58570700 | 0.22424700 |
| C | -0.70607300 | -0.60579500 | -1.28185500 |
| C | 0.64155400 | -1.33230100 | -1.03259100 |
| C | -0.92213400 | -0.38168000 | 1.32571400 |
| C | 0.27003000 | -1.03859400 | 1.36756100 |
| H | -0.52477200 | 0.14330900 | -2.06020600 |
| H | -1.55305200 | -1.22263800 | -1.61664300 |
| H | 0.50447100 | -2.39746800 | -0.82150300 |
| H | 1.32428400 | -1.22429200 | -1.87945400 |
| H | -1.70285500 | -0.29985100 | 2.08448700 |
| H | 0.50374500 | -1.92770000 | 1.95102700 |
| C | 1.50765600 | 0.74144000 | 0.11004400 |
| N | 1.94846700 | 1.81692900 | -0.01536100 |
| N | -1.82086100 | 1.26892600 | -0.18971800 |
| H | -2.00690000 | 1.88985800 | 0.58345200 |
| H | -1.92789500 | 1.66439100 | -1.11114200 |
